# Validity and reliability of EQ-5D-3L for breast cancer patients in Korea

**DOI:** 10.1186/s12955-015-0399-x

**Published:** 2015-12-23

**Authors:** Seon-Ha Kim, Min-Woo Jo, Jong-Won Lee, Hyeon-Jeong Lee, Jong Kyung Kim

**Affiliations:** Department of Nursing, Dankook University, Cheonan, South Korea; Department of Preventive Medicine, University of Ulsan College of Medicine, 88, Olympicro 43-gil, Songpa-gu, 138-736 Seoul South Korea; Department of Breast and Endocrine Surgery, Asan Medical Center, Seoul, South Korea; Department of Surgery, University of Ulsan College of Medicine, Seoul, South Korea

**Keywords:** Breast cancer, EQ-5D-3L, Health-related quality of life, FACT-B, Korea

## Abstract

**Background:**

Recently, breast cancer incidence and prevalence has been increasing. Patients' health related quality of life is important considerations in the treatment of breast cancer. The EQ-5D-3L is one of most popular instruments to measure health related quality of life. This study was aimed to evaluate the validity and reliability of EQ-5D-3L in post-operative breast cancer patients from Korea.

**Methods:**

A total of 827 patients visiting the ambulatory cancer center of 1 tertiary hospital after breast cancer surgery self-administered the EQ-5D-3L and Functional Assessment of Cancer Therapy-Breast Cancer (FACT-B). We evaluated known-group validity using differences in the EQ-5D-3L index and EQ-VAS score according to demographic and clinical data. The discriminatory ability of the EQ-5D-3L was determined by comparing the mean FACT-B subscale scores between subjects with no problems and subjects with moderate or severe problems in each EQ-5D-3L dimension. Construct validity was evaluated by Pearson correlation coefficients among the EQ-5D-3L index and FACT-B subscales, respectively. Reliability was assessed in terms of test-retest reliability using Cohen’s kappa value and intra-class correlation coefficient (ICC).

**Results:**

The EQ-5D-3L index and EQ-VAS score were higher in the educated, current radiotherapy and unmarried groups. The correlation of EQ-5D-3L index and subscales for the FACT-B was highest in physical well-being (r = 0.553) and lowest in social well-being (r = 0.199). For reliability, the Kappa values’ range was from 0.32 to 0.70, and ICCs of the EQ-5D-3L index and EQ-VAS scores were 0.70 and 0.48, respectively.

**Conclusions:**

This study indicated that the EQ-5D-3L could be a valid health related quality of life instrument for postoperative breast cancer patients.

## Background

Breast cancer is the most frequently diagnosed cancer in females and the leading cause of cancer-related deaths in both economically developed and developing countries [1]. In Korea, the crude breast cancer incidence rate was 63.7 per 100,000 persons in 2011 and has been increasing since 1999 [2]. The 5-year survival rate for breast cancer patients has improved, with 91.3 % reported between 2007 and 2011 in Korea [2]. Recent years have led to a range of treatments for breast cancer, and development of additional therapeutics is continually in progress.

Health-related quality of life (HRQoL) and traditional clinical outcomes (i.e., survival rate or tumor responses) are considered as significant outcomes of cancer care [3]. Tools to assess HRQoL are classified into disease-specific instruments and generic instruments. Preference-based instruments (the Health Utility Index [4, 5], the EuroQol 5D (EQ-5D-3L) [6], or the Short Form 6D [7]) are generic instrument generating utilities that yield a measure that combines both the length and quality of life [8]. Disease-specific instruments can be more sensitive for detecting clinically important differences or changes in specific disease groups in general, although general instrument is occasionally not inferior to disease-specific instrument [9]. However, they do not compare HRQoL of patients with other diseases [8]. Resource allocation for health care has been a critical issue, not only for its effectiveness, but also for efficiency of care; hence, it is important for guiding healthcare policy.

The EQ-5D-3L, a generic preference-based instrument that is widely used to measure HRQoL, can be used to assign preference values to various health states [10]. Additionally, EQ-5D-3L is a unique instrument with quality weight tariffs for use in Korean populations [11]. The psychometric properties of the Korean EQ-5D-3L have been studied for rheumatic disease [12] and colon cancer [13] patients. However, studies on validity and reliability of the EQ-5D-3L in breast cancer patients in Korea are scarce [14]. Hence, we evaluated the validity and reliability of the EQ-5D-3L to measure the HRQoL in post-operative breast cancer patients from Korea.

## Methods

### Subjects and settings

A consecutive series of breast cancer patients who had surgery as a primary treatment at the ambulatory cancer center of 1 tertiary hospital in Seoul, Korea from February 2012 to May 2012, participated in this study. All participants provided informed written consent prior to taking our survey and a total of 1,002 subjects were consecutively recruited. The initial 150 recruited subjects were requested to complete an EQ-5D-3L questionnaire 1 week later via mail to assess the test-retest reliability of each patient. The survey was self-administered in the waiting room at the center with or without research nurse assistance. The Institutional Review Board of Asan Medical Center approved the study (IRB approval number: 2012–0010).

### Information

Our questionnaire included requests for demographic information (age, sex, level of education, marital status, and occupation) and 2 HRQOL instruments (EQ-5D-3L and Functional Assessment of Cancer Therapy-Breast Cancer [FACT-B] version 4). Clinical information (the type of surgery, American Joint Committee on Cancer [AJCC] 7^th^ stage at diagnosis, duration of disease since diagnosis, and current treatment for breast cancer), was obtained from the cancer registry database of the center.

The EQ-5D-3L is an instrument widely used to measure and evaluate the general health status of a patient in 5 dimensions (mobility, self-care, usual activities, pain/discomfort, and anxiety/depression) with 3 levels as follows: 1, no problems; 2, some or moderate problems; and 3, extreme problems. The EQ-5D-3L provides a simple descriptive profile and a single index of health status [10]. The EQ**-**VAS records the respondent’s self-rated health on a vertical, visual analogue scale where the endpoints are classified as follows: ‘best imaginable health state’ =100 and ‘worst imaginable health state’ =0 [10].

The FACT-B is a validated multi-dimensional self-reported questionnaire with a 36-item questionnaire that measures both the 27-item general QoL associated with cancer (Functional Assessment of Cancer Therapy - General [FACT-G]) and an additional 9-item breast cancer-related QOL. The FACT-B includes the following subscales that measure physical well-being (PWB), functional well-being (FWB), emotional well-being (EWB), social/family well-being (SWB), and the breast cancer subscale (BCS). FACT-B total scores were calculated as the sum of the 5 subscales. FACT-G scores were calculated as the sum of the PWB, FWB, EWB, and SWB scores and FACT-B Trial Outcome Index (TOI) was calculated as the addition of the PWB, FWB and BCS scores [15, 16]. This study used the Korean FACT-B version 4 that is validated [17] and a higher score indicated a better state.

### Analyses

The proportion of patients reporting any problems in each EQ-5D-3L dimension and EQ-5D-3L index were presented. The EQ-5D-3L index was calculated using a Korean valuation set [11]. The EQ-5D-3L index ranged from −0.171 to 1, and higher values indicated better health status. The FACT-B subscale scores were calculated in accordance with a scoring guideline provided by the Functional Assessment of Chronic Illness Therapy (FACIT) measurement system (www.facit.org/FACITOrg).

Differences in the EQ-5D-3L index and EQ-VAS score according to demographic and clinical data were compared using the Student’s *t*-test or analysis of variance (ANOVA). In aspects of known group validity, we expected that the EQ-5D-3L index and EQ VAS would be lower for the following conditions: worse stage at diagnosis [18], current treatment group [18–20], shorter duration of disease since diagnosis [19, 21–24], older age group [16, 22], and lower education group [16, 18, 22].

The discriminatory ability of the EQ-5D-3L was determined by comparing the mean FACT-B subscale scores between subjects with no problems and subjects with moderate or severe problems in each EQ-5D-3L dimension. It was expected that the FACT-B subscale scores of subjects with no problem would be higher than those of subjects with any problem. The mean differences in the FACT-B subscale were compared using the Student’s *t*-test. Discriminatory ability of the EQ-5D-5 L was considered acceptable when all hypothesized differences were significant.

Construct validity was evaluated by Pearson correlation coefficients among the EQ-5D-3L index, EQ-VAS, and FACT-B subscales. EQ-5D-3L index and FACT-G total scores were expected to be moderately or strongly correlated, because both instruments measured HRQoL. It was expected that the correlations between the EQ-5D-3L index and SWB subscale would be weaker than those of the other FACT-B subscales, because the EQ-5D-3L does not contain a social dimension. Construct validity was considered as acceptable when the hypotheses were satisfied.

Cohen’s kappa statistic was used to assess the test-retest reliability of each dimension for the EQ-5D-3L instrument. The kappa statistic calculates the degree of agreement in classification over that which would be expected by chance. There is no universally agreed-upon criteria of significant kappa value. We considered test-retest reliability of EQ-5D-5 L as acceptable if kappa value was higher than 0.4 in each dimension [25]. The intraclass correlation coefficient (ICC) [26] was used to assess the reliability of the EQ-5D-3L index and EQ-VAS score. We considered that > 0.5 ICC was acceptable.

## Results

Among 1,002 recruited patients with breast cancer, 175 patients were excluded from this analysis: 13 duplicated, 2 with missing EQ-5D-3L data, and 160 patients who did not merge into the cancer registry data. Thus, a total of 827 patients were included in the study cohort. Among 150 participants who completed a second survey 1 week later, 67 participants replied and 54 subjects were used for test-retest reliability after excluding 13 participants for whom clinical information was not available. Demographic characteristics of the survey participants were shown in Table 1. All study participants were female with a mean age of 52.1 years. Most patients were married. The number of participants who had undergone breast-conserving surgery was 524 (63.4 %).

**Table 1:** General and clinical characteristics of the study subjects

Variables	N	(%)
Age, years		
Less than 40	54	(6.5)
40–49	263	(31.8)
50–59	385	(46.6)
60 or more	125	(15.1)
Level of education		
Elementary school	36	(4.4)
Middle school graduate	80	(9.7)
High school graduate	338	(40.9)
College or higher	371	(44.9)
Marital status		
Married	692	(83.7)
Widowed	45	(5.4)
Divorced	39	(4.7)
Unmarried	51	(6.2)
Surgery		
Mastectomy	303	(36.6)
Breast conserving surgery	524	(63.4)
AJCC 7^th^ stage at diagnosis		
0	136	(16.5)
1	361	(43.8)
2	260	(31.5)
3	68	(8.2)
Current treatment		
Chemotherapy	26	(3.2)
Radiotherapy	55	(6.7)
Duration of disease since diagnosis		
<1 year	147	(17.8)
1 - < 2 years	104	(12.6)
2 - < 3 years	106	(12.8)
3 - < 4 years	125	(15.1)
4 - < 5 years	91	(11.0)
≥5 years	254	(30.7)

The proportion of patients reporting any problems of the EQ-5D-3L and the FACT-B subscale score distribution in the study participants was shown in Table 2. The proportion of patients reporting any problems was relatively higher in the pain/discomfort and anxiety/depression dimensions at 43.5 % and 41.5 %, respectively. The mean FACT-G score was 78.7 (SD 15.8) and mean TOI was 66.7 (12.3). The EQ-5D-3L index and VAS score according to demographic and clinical features were shown in Table 3. In the highly educated and unmarried group, and not in current radiotherapy group, the EQ-5D-3L index scores were higher than corresponding scores of the other groups. A worse stage at the time of diagnosis was associated with lower HRQoL scores; however, this trend did not reach the threshold for statistical significance. By post-hoc analysis, individuals with < 1 year since the time of diagnosis had a significantly lower EQ-5D-3L index as compared with those who had > 4 years since their diagnosis. The EQ-VAS score were not significantly different according to demographic and clinical characteristics.

**Table 2:** Distribution of EQ-5D-3L problem reporting and FACT-B scores

EQ-5D-3L dimension	Level^a^	N	(%)
Mobility	1	760	(91.9)
	2	67	(8.1)
	3	0	(0.0)
Self-care	1	791	(95.7)
	2	33	(4.0)
	3	3	(0.4)
Usual activities	1	720	(87.1)
	2	105	(12.7)
	3	2	(0.2)
Pain/discomfort	1	467	(56.5)
	2	351	(42.4)
	3	9	(1.1)
Anxiety/depression	1	484	(58.5)
	2	334	(40.4)
	3	0	(1.1)
FACT-B scale scores (possible range)		Mean	(SD)
Physical well-being (0–28)		24.0	(4.3)
Social well-being (0–28)		17.0	(6.9)
Functional well-being (0–28)		19.3	(6.4)
Emotional well-being (0–24)		18.3	(4.0)
Breast cancer subscale (0–36)		23.3	(5.4)
FACT-G (0–108)		78.7	(15.8)
FACT-B Trial Outcome Index (0–92)		66.7	(12.3)
FACT-B total score (0–144)		102.1	(19.0)

*FACT-B* functional assessment of cancer therapy—Breast cancer

*FACT-G* functional assessment of cancer therapy—General

Higher scores on the FACT-B scales indicate better quality of life

^a^1: no problem; 2: moderate problems; 3: extreme problems

**Table 3:** EQ-5D-3L scores according to general and clinical characteristics of the study patients

Variables		EQ-5D-3L index			EQ-VAS		
(*n* = 827)	n	M	SD	p-value^*^	M	SD	p-value^*^
Age group (year)							
Less than 40	54	0.918	(0.071)	0.448	76.3	(18.0)	0.338
40–49	263	0.921	(0.075)		77.8	(17.5)	
50–59	385	0.912	(0.092)		78.5	(16.3)	
60 or more	125	0.907	(0.105)		80.6	(15.8)	
Level of education (year)							
≤6	36	0.864	(0.099)	<0.001	81.9	(16.0)	0.629
7–9	80	0.880	(0.100)		77.7	(16.8)	
10–12	338	0.916	(0.090)		78.2	(17.9)	
≥13	371	0.925	(0.079)		78.4	(15.8)	
Marital status							
Married	692	0.917	(0.086)	0.045	78.6	(16.7)	0.697
Widowed	45	0.901	(0.097)		79.0	(15.4)	
Divorced	39	0.879	(0.125)		78.1	(20.0)	
Unmarried	51	0.921	(0.072)		75.7	(16.2)	
Surgery							
Mastectomy	303	0.915	(0.088)	0.872	78.8	(16.9)	0.678
Breast conserving surgery	524	0.914	(0.088)		78.2	(16.6)	
AJCC 7^th^ stage at diagnosis							
0	136	0.925	(0.081)	0.083	78.2	(16.3)	0.929
1	361	0.918	(0.093)		78.1	(16.6)	
2	260	0.909	(0.083)		79.0	(16.4)	
3	68	0.895	(0.088)		78.4	(19.1)	
Current chemotherapy							
No	793	0.915	(0.088)	0.361	78.5	(16.6)	0.602
Yes	26	0.899	(0.081)		76.7	(18.9)	
Current radiotherapy							
No	764	0.917	(0.087)	0.001	78.8	(16.3)	0.047
Yes	55	0.877	(0.102)		72.8	(21.0)	
Duration of disease since diagnosis							
<1 year	147	0.893	(0.090)	0.003	77.5	(18.1)	0.385
1 - < 2 years	104	0.903	(0.084)		76.7	(16.7)	
2 - < 3 years	106	0.921	(0.093)		78.2	(17.5)	
3 - < 4 years	125	0.914	(0.090)		78.5	(16.1)	
4 - < 5 years	91	0.934	(0.066)		81.8	(13.9)	
≥5 years	254	0.922	(0.090)		78.6	(16.9)	

^*^ *p* < 0.05 by Student’s *t*-test or ANOVA

FACT-B subscale scores and summary measurements by dimension and level of the EQ-5D-3L were shown in Table 4. All FACT-B subscale scores and combined index of subjects with no problem were significantly higher than those of subjects with any problem in all EQ-5D-3L dimensions. Difference in PWB scores was larger than other FACT-B subscales in mobility dimension, whereas difference in PWB scores was larger in self-care and usual activities dimensions. Difference in EWB scores was the largest in the anxiety/depression dimension. Table 5 showed correlation coefficients among the EQ-5D-3L index, EQ-VAS score, and FACT-B scores. TOI scores showed the highest correlation with both the EQ-5D-3L index and EQ-VAS scores at 0.557 and 0.456, respectively, and the SWB scores showed the lowest correlation with the EQ-5D-3L index. Rates of agreements in the EQ-5D-3L dimensions ranged from 85.2 to 96.3 % across dimensions. Kappa statistics ranged from 0.32 in mobility to 0.70 in pain/discomfort and anxiety/depression dimensions. ICCs of the EQ-5D-3L index and EQ-VAS scores were 0.70 and 0.48, respectively (Table 6).

**Table 4:** FACT-B scales and summary measurements by EQ-5D-3L dimension and level

Dimension	Level^a^	n	PWB	SWB	EWB	FWB	FACTG	BCS	TOI	FACT-B total
Mobility	1	760	24.4^**^	17.1^**^	18.5^**^	19.6^**^	79.7^**^	23.7^**^	67.7^**^	103.4^**^
	2 or 3	67	19.7	14.9	16.8	16.1	67.6	19.6	55.5	87.3
Self-care	1	791	24.2^**^	17.1^*^	18.4^*^	19.6^**^	79.4^**^	23.5^**^	67.3^**^	102.9^**^
	2 or 3	36	20.0	14.1	17.0	13.7	64.0	20.5	53.5	84.4
Usual activities	1	720	24.7^**^	17.3^**^	18.6^**^	20.0^**^	80.7^**^	23.8^**^	68.5^**^	104.5^**^
	2 or 3	107	19.8	14.6	16.3	14.5	65.4	20.0	54.4	85.5
Pain/discomfort	1	467	25.4^**^	17.5^*^	18.9^**^	20.4^**^	82.3^**^	24.4^**^	70.2^**^	106.7^**^
	2 or 3	360	22.2	16.3	17.6	17.9	74.1	22.0	62.1	96.0
Anxiety/depression	1	484	25.3^**^	18.0^**^	20.0^**^	21.2^**^	84.6^**^	24.9^**^	71.4^**^	109.5^**^
	2 or 3	343	22.2	15.5	15.9	16.6	70.4	21.1	60	91.6

*FACT-B* functional assessment of cancer therapy—Breast cancer, *FACT-G* functional assessment of cancer therapy—general, *PWB* physical well-being, *FWB* functional well-being, *EWB* emotional well-being, *SWB* social/family well-being (SWB), *BCS* breast cancer subscale, *TOI* Trial outcome index

^a^1,no problem; 2,moderate problems; 3,extreme problems

^*^ *p* < 0.05 by Student’s *t*-test

^**^ *p* < 0.001 by Student’s *t*-test

**Table 5:** Correlations between the EQ-5D-3L index, EQ-VAS score, and FACT-B scores

FACT-B scores	EQ-5D-3L index	EQ VAS
Physical well-being	0.553	0.447
Social well-being	0.199	0.170
Emotional well-being	0.400	0.371
Functional well-being	0.377	0.312
Breast cancer subscale	0.390	0.323
FACTG	0.488	0.414
Trial Outcome Index	0.557	0.456
FACT B total	0.516	0.436

*FACT-B* functional assessment of cancer therapy—breast cancer, *FACT-G* functional assessment of cancer therapy—general

Pearson’s correlation coefficient, all p-values were <0.001

**Table 6:** Test-retest reliability of the EQ-5D-3L

Dimension	Kappa statistics (95 % CI) all patients (n = 54)	Agreement rate (%)
Mobility	0.32 (−0.18–0.93)	94.4
Self-care	0.49 (−0.11–1.00)	96.3
Usual activities	0.46 (0.13–0.80)	87.0
Pain/discomfort	0.70 (0.51–0.89)	85.2
Anxiety/depression	0.70 (0.52–0.89)	85.2
Intraclass correlation coefficient (95 % CI)		
EQ-5D-3L index	0.70 (0.54–0.82)	
EQ-VAS	0.48 (0.25–0.66)	

## Discussion

In this study, we aimed to assess the validity and reliability of EQ-5D-3L for measuring HRQoL in post-operative breast cancer patients. Our findings supported the validity of the EQ-5D-3L index. Values for this index were lower in the current radiation therapy, shorter duration of disease since diagnosis, and low education groups. Most of known-group validity features fulfilled our hypothesis. EQ-5D-3L indexes were lower for progressive stages at diagnosis and in the current chemotherapy group, although the differences in the EQ-5D-3L index between groups were not statistically significant. Our results corroborated the findings of Chae and Seo that patients who are younger, more highly educated, and with a longer time since diagnosis have a higher HRQoL [22]. HRQoL score differences by age group were not consistent with previous reports of HRQoL measurements in breast cancer patients [16, 22, 23]. Age has different impacts on HRQoL domains. Ahn et al’s study on breast cancer survivors showed better social and emotional function but poorer physical function with increasing age [24].

The FACT-B subscale scores for problem reporting in each EQ-5D-3L dimension were significantly different in the current analysis. The EQ-5D-3L index was moderately correlated with FACT-B subscale scores, ranging from 0.377 at FWB to 0.553 at PWB subscales, except for SWB. This finding was similar to those of Lee et al. who studied patients with breast cancer in Singapore [26]. In that study, a correlation between the EQ-5D-3L index and FACT-B subscales was the lowest at 0.11 on the SWB subscales and the highest at 0.66 on the PWB subscales [26].

Reliability of EQ-5D-3L in this study was inconclusive. Kappa values for EQ-5D-3L dimensions varied depending on domain and confidence intervals of kappa values were wide in mobility, self-care and usual activities. The ICCs of the EQ-5D-3L index was reasonable but EQ-VAS were somewhat low. Kappa values on mobility dimensions were the lowest at 0.32, but showed some agreement with the Landis and Koch criteria [25] at an agreement rate of 94.4 %. Low reliability statistics could be due to small sample size, change of health states between 2 time periods, and high proportion of no problem in mobility, self-care and usual activities dimensions. In the previous Singapore study, ICCs for the EQ-5D-3L index were reported to as 0.81 in the English version and 0.69 in the Chinese version [27]. Similarly, another Korean study of colorectal cancer patients reported kappa values for the EQ-5D-3L dimensions ranging from 0.32 to 0.56 and the ICC of the EQ-5D-3L index of 0.55 [13]. Compared to our present study, an earlier Taiwan study on cervical cancer patients reported somewhat higher kappa values for the EQ-5D-3L dimensions and the ICC of the EQ-5D-3L index [28].

The findings of the present study cannot be generalized since only postoperative breast cancer patients at 1 tertiary hospital were enrolled. Another limitation was that we did not investigate patient recurrence status, time since treatment completion, performance status at the visit day, or comorbidity. Because these parameters could affect the HRQoL, more comprehensive data are needed in the future to assess the psychometric properties of the HRQoL instruments. In addition, we did not assess the responsiveness of the EQ-5D-3L in breast cancer interventions; hence, further studies are warranted. Lastly, we did not reassess physical status at retest period, thus evidence on test-retest reliability was inconclusive.

## Conclusion

In conclusion, we showed that the EQ-5D-3L could be a valid instrument for measuring the HRQoL of post-operative breast cancer patients in South Korea. However, reliability of the EQ-5D-3L is not conclusive and needs further research.

## Abbreviations

ANOVA: analysis of variance
BCS: breast CANCER subscale
EWB: emotional well-being
FACT-B: Functional Assessment of Cancer Therapy-Breast Cancer
FACT-G: Functional Assessment of Cancer Therapy - General
FWB: Functional well-being
HRQoL: health-related quality of life
ICC: intra-class correlation coefficient
PWB: physical well-being
SWB: social/family well-being
